# High-Sensitivity Cardiac Troponin T in Patients with Severe Chronic Kidney Disease and Suspected Acute Coronary Syndrome

**DOI:** 10.3390/jcm10184216

**Published:** 2021-09-17

**Authors:** Brunilda Alushi, Fabian Jost-Brinkmann, Adnan Kastrati, Salvatore Cassese, Massimiliano Fusaro, Karl Stangl, Ulf Landmesser, Holger Thiele, Alexander Lauten

**Affiliations:** 1 Department of Cardiovascular Diseases, Campus Benjamin Franklin, Charité—Universitätsmedizin Berlin, Corporate Member of Freie Universität Berlin and Humboldt-Universität zu Berlin, Hindenburgdamm 30, 12203 Berlin, Germany; fabian.jost-brinkmann@charite.de (F.J.-B.); ulf.landmesser@charite.de (U.L.); alexander.lauten@helios-gesundheit.de (A.L.); 2 German Centre for Cardiovascular Research (DZHK), Partner Site Berlin, Potsdamer Str. 58, 10785 Berlin, Germany; karl.stangl@charite.de; 3 Department of General and Interventional Cardiology, Helios Klinikum Erfurt, Nordhäuser Str. 74, 99089 Erfurt, Germany; 4 Department of Hepatology and Gastroenterology, Campus Virchow Klinikum (CVK) and Campus Charité Mitte (CCM), Charité—Universitätsmedizin Berlin, Corporate Member of Freie Universität Berlin and Humboldt-Universität zu Berlin, Augustenburger Platz 1, 13353 Berlin, Germany; 5 German Heart Center Munich, Technische Universität München, Lazarettstraße 36, 80636 Munich, Germany; kastrati@dhm.mhn.de (A.K.); cassese@dhm.mhn.de (S.C.); massimiliano.fusaro@artemed.de (M.F.); 6 German Centre for Cardiovascular Research (DZHK), Partner Site Munich Heart Alliance, 80336 Munich, Germany; 7 Department of Cardiovascular Diseases, Campus Charité Mitte (CCM), Charité—Universitätsmedizin Berlin, Charitéplatz 1, 10117 Berlin, Germany; 8 Berlin Institute of Health at Charité—Universitätsmedizin Berlin, Charitéplatz 1, 10117 Berlin, Germany; 9 Department of Internal Medicine/Cardiology, Heart Center Leipzig at University of Leipzig, Strümpellstraße 39, 04289 Leipzig, Germany; holger.thiele@medizin.uni-leipzig.de; 10 Leipzig Heart Institute, Russenstraße 69a, 04289 Leipzig, Germany

**Keywords:** troponin, high-sensitivity, chronic kidney disease, renal insufficiency, myocardial infarction, acute coronary syndrome

## Abstract

(1) Background: Patients with severe chronic kidney disease (CKD G4–G5) often have chronically elevated high-sensitivity cardiac troponin T (hs-cTnT) values above the 99th percentile of the upper reference limit. In these patients, optimal cutoff levels for diagnosing non-ST-elevation acute coronary syndrome (NSTE-ACS) requiring revascularization remain undefined. (2) Methods: Of 11,912 patients undergoing coronary angiography from 2012 to 2017 for suspected NSTE-ACS, 325 (3%) had severe CKD. Of these, 290 with available serial hs-cTnT measurements were included, and 300 matched patients with normal renal function were selected as a control cohort. (3) Results: In the CKD cohort, 222 patients (76%) had NSTE-ACS with indication for coronary revascularization. Diagnostic performance was high at presentation and similar to that of the control population (AUC, 95% CI: 0.81, 0.75–0.87 versus 0.85, 0.80–0.89, *p* = 0.68), and the ROC-derived cutoff value was 4 times higher compared to the conventional 99th percentile. Combining the ROC-derived cutoff levels for hs-cTnT at presentation and absolute 3 h changes, sensitivity increased to 98%, and PPV and NPV improved up to 93% and 86%, respectively. (4) Conclusions: In patients with severe CKD and suspected ACS, the diagnostic accuracy of hs-cTnT for the diagnosis of NSTE-ACS requiring revascularization is improved by using higher assay-specific cutoff levels combined with early absolute changes.

## 1. Introduction

Acute myocardial infarction (AMI) is a leading cause of death and disability worldwide. In the setting of acute coronary syndrome, rapid and accurate identification of patients requiring revascularization is crucial to initiate evidence-based therapy without causing unnecessary harm [1,2,3]. One third of the patients presenting with persistent ST-segment elevation myocardial infarction (STEMI) and more than 40% of patients with non-ST-elevation acute myocardial infarction (NSTEMI) have chronic kidney disease (CKD) [4].

The early diagnosis and therapy of NSTE-ACS in this population can be challenging, mostly due to frequent atypical clinical presentation, preexisting electrocardiogram abnormalities, and the vulnerability of these patients to adverse events related to antiplatelet treatment and invasive procedures as coronary interventions [5,6]. These patients, especially those undergoing dialysis for kidney failure (CKD G5D), are therefore less likely to receive guideline-indicated care despite several studies indicating a higher risk of poor outcomes after AMI [3,4,7]. In particular, coronary angiography is performed too infrequently in the context of NSTEMI-ACS in patients with CKD [3,8,9,10,11].

Cardiac-specific biomarkers, such as high-sensitivity troponin T (hs-cTnT) and troponin I (hs-cTnI), are the cornerstone in diagnosing myocardial infarction (MI) irrespective of renal function [2,12]. However, their clinical utility in patients with renal dysfunction is a matter of concern since the values of hs-cTnT are frequently chronically elevated in the presence of CKD even in absence of AMI [13,14,15]. The identification of cutoff values of hs-cTnT for diagnosis of NSTEMI is based on healthy populations; thus, the optimal cutoff level in CKD patients is probably higher [16,17]. Few studies investigating the optimal cutoffs of hs-cTnT levels in patients with CKD reported higher cutoffs with a lower specificity and overall accuracy compared to the healthy population [17,18,19,20]. However, the most vulnerable CKD patients, those with CKD G4 (eGFR 29–15 mL/min/1.73 m^2^), were constantly underrepresented or, as in case of patients with CKD G5 (eGFR < 15 mL/min/1.73 m^2^) and kidney failure treated by dialysis (CKD G5D), even excluded from most studies [17,19,20,21,22]. We therefore aimed to investigate the diagnostic performance and identify the optimal cutoff of hs-cTnT for the diagnosis of NSTE-ACS requiring revascularization in patients with severe CKD, including those with KFRT, by using coronary angiography as reference.

## 2. Materials and Methods

### 2.1. Study Design and Data Collection

This observational study comprised patients with severe CKD undergoing coronary angiography for suspected ACS at three tertiary cardiovascular centers of the Charite’ University Hospital in Berlin from January 2012 to December 2017 and who were prospectively included in the local MI registry. The study complies with the Declaration of Helsinki and was approved by the locally appointed ethics committee.

**Figure 1:**
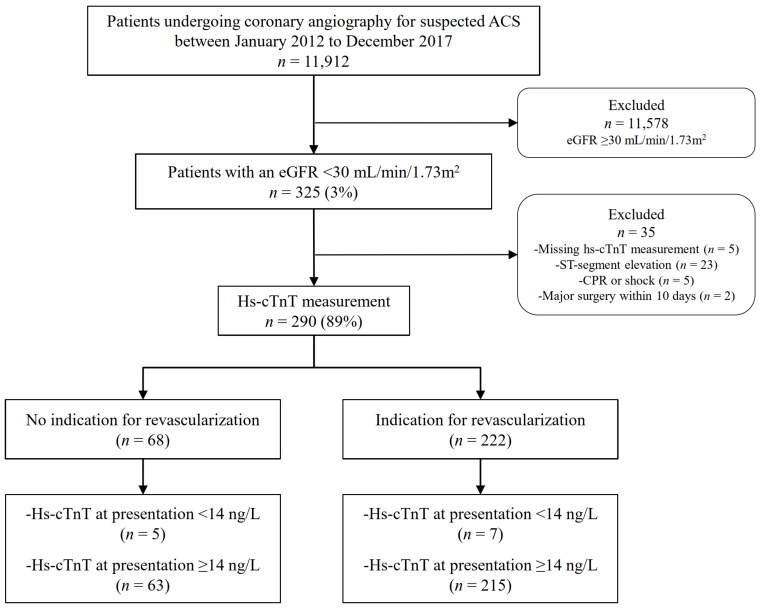
Flow chart of patient selection process. ACS, acute coronary syndrome; CPR, cardiopulmonary resuscitation; ED, emergency department; eGFR, estimated glomerular filtration rate according to the Chronic Kidney Disease Epidemiology Collaboration (CKD-EPI) formula.

Among all unselected patients undergoing coronary angiography for suspected ACS, those with severe CKD and at least one available hs-cTnT measurement were identified. Severe CKD was defined as an eGFR < 30 mL/min/1.73 m^2^ in the presence of a known history of CKD, as previously described [23]. CKD G5D was defined by the need for long-term dialysis for at least 30 days [24,25]. Two independent cardiologists (BA and AL) a-posteriori adjudicated the final diagnosis of NSTE-ACS and the indication for revascularization after reviewing all available medical records, including patient history, physical examination, results of laboratory testing, electrocardiograms, echocardiographs, cardiac exercise tests, and coronary angiograms obtained from the time of the index event to one year of follow-up. If there was disagreement about the clinical diagnosis or the need for revascularization, the cases were reviewed and decided collaboratively. AMI was classified according to the latest definition of the Task Force for the universal definition of MI [2]. In patients with NSTE-ACS, the indication for revascularization with either percutaneous coronary intervention (PCI) or coronary artery bypass graft (CABG) was adjudicated based on the severity and morphology of the lesion on coronary angiogram in correlation with clinical, laboratory, electro-, and echocardiographic parameters, as recommended by current ESC guidelines [1,3]. Accordingly, patients were stratified to either the revascularization group undergoing PCI or CABG or to the non-revascularization group, receiving medical treatment only. For this study, patients with STEMI were excluded. Patients were also excluded for missing hs-cTnT measurements, symptom onset or peak not within the last 12 h from presentation, cardiopulmonary resuscitation, shock at the time of presentation, or undergoing previous PCI or major surgery within ten days prior to hospital admission. Figure 1 depicts the patient selection process. For the purpose of this study, patients with NSTEMI type 2 (coronary vasospasm, tachy- or bradyarrhythmias, and hypertensive crisis) or other conditions that may cause myocardial injury with hs-cTnT elevations, such as pulmonary embolism, endo-/myocarditis, non-ischemic congestive heart failure, or Takotsubo cardiomyopathy, were excluded from the final receiver operating characteristic curve (ROC) analysis. The study was conducted according to the STARD guidelines (Supplementary Figure S1).

Patients with normal renal function (defined as an eGFR > 90 mL/min/1.73 m^2^) with available hs-cTnT measurements and who were undergoing coronary angiography for suspected NSTE-ACS were selected as a control cohort. A matched random sampling on the baseline characteristics gender, age, coronary artery disease (CAD), arterial hypertension, and chronic obstructive pulmonary disease (COPD) was used to equate the control with the CKD cohort. At each site, data were collected at admission, discharge, and scheduled follow-up at one year. Any clinical event since hospital discharge was collected by reviewing electronic patient records of the hospitals and family physicians or by telephone contact with the patient or their family.

### 2.2. Clinical and Laboratory Assessment

All data from the clinical assessment, including physical examination, 12-lead electrocardiogram, pulse oximetry, standard blood test, and medical history, were obtained from medical records. Baseline characteristics, known cardiovascular risk factors and comorbidities, were recorded. Renal function was quantified by eGFR, as recommended, using the Chronic Kidney Disease Epidemiology Collaboration (CKD-EPI) formula based on plasma creatinine levels obtained at the index event, age, sex, and ethnicity, as previously described [23]. Blood samples for determination of hs-cTnT at presentation, 3 h, 6 h, and peak prior to coronary angiography from symptom onset were collected into tubes containing potassium EDTA- or lithium-heparinized plasma [26]. Hs-cTnT was measured using a fifth-generation electrochemiluminescence assay (Elecsys, Cobas e602 analyzer, Roche Diagnostics). According to the manufacturer, the 99th percentile of the upper reference limit (URL) in healthy individuals is 14 ng/L, the coefficient of variation < 10% at 13 ng/L, and the limit of detection is 5 ng/L [16]. For patients undergoing dialysis, only blood tests during the dialysis interval were collected.

### 2.3. Outcomes

The primary diagnostic outcome was the identification of an optimal cutoff of hs-cTnT for the diagnosis of NSTE-ACS, requiring revascularization either by PCI or CABG. Prognostic outcomes were all-cause mortality, cardiovascular mortality, MI, and major adverse cardiovascular events (MACE), defined as the composite of cardiovascular death, MI, or unplanned revascularization for ischemia within one year. Survival time for each outcome was defined as the period from the date of the index coronary intervention in the intervention group or from the date of the index presentation in the conservative treatment group to the first event during the one-year follow-up. For all analyses, a comparison to the matched control cohort with normal renal function was performed. Subgroup analyses were conducted for patients with CKD G5D and compared to patients with CKD G4–G5.

### 2.4. Statistical Analysis

Data are expressed as median with interquartile range (IQR) or frequencies and percentages. All variables were tested for normal distribution with the Shapiro–Wilk test. Discrete variables were compared by using Fisher’s exact test and continuous ones with the Wilcoxon rank-sum test for independent samples. The hs-cTnT changes from presentation to peak according to revascularization groups were analyzed overall and within each group using a linear mixed model considering the within-patient correlation. Correlations between renal function and levels of hs-cTnT were assessed by Spearman’s rank correlation test. Area under the ROC (AUC) was calculated to assess the diagnostic performance of hs-cTnT at each measured time point. Logistic regression was used to combine hs-cTnT levels at presentation with early absolute changes in hs-cTnT levels. Comparison of diagnostic accuracy was also performed according to the presence or absence of ESRD. The comparison of AUC was carried out as recommended by DeLong et al. for dependent samples and by Hanly and McNeil for independent samples [27,28]. The optimal ROC-derived cutoff levels were identified using the Youden index and compared to the 99th percentile of healthy individuals as well as cutoff levels that achieve predefined sensitivities (>90%) and specificities (>80%). Negative predictive value (NPV) and positive predictive value (PPV) were calculated as prevalence-dependent parameters. The decision to use the 99th percentile of the URL in healthy individuals was guided by its use in guideline recommendations for the diagnosis of AMI [12].

Kaplan–Meier analysis estimated the cumulative incidence of outcomes at 30 days and one year. Adjusted hazard ratios (HRs) were calculated in multivariable Cox regressions, including baseline characteristics known to impact patient’s outcome. Baseline variables with *p* < 0.10 at the univariate analysis were included in the model. Results are presented as hazard ratios (HRs) and 95% confidence intervals (95% CI). The proportional assumptions hazard was tested based on Schoenfeld residuals. All probability values were 2-tailed and considered significant when *p* < 0.05. Data analysis was performed using STATA15.0 (StataCorp, College Station, TX, USA).

## 3. Results

### 3.1. Baseline and Periprocedural Characteristics

Among 11,912 unselected patients undergoing coronary angiography for suspected AMI, 325 (3%) had severe CKD, and of these, 290 (89%) with an available measurement of hs-cTnT at the time of presentation were included in the study. Serial hs-cTnT measurements were available in 243 patients (84%) at 3 h, 6 h, and/or during follow-up until coronary angiography. The most common reason for missing samples at 3 and 6 h was early transfer to the catheter laboratory. The control cohort included 300 matched patients with normal renal function undergoing coronary angiography for suspected NSTE-ACS.

Of the 290 patients with severe CKD, 222 (77%) had an a-posteriori adjudicated indication for revascularization via PCI (89%) or CABG (11%), and 68 (23%) had no need for coronary revascularization. In the control population with normal renal function, 72% of the patients required immediate revascularization (*p* = 0.156) with similarly distributed percentages of PCI and CABG compared to the cohort with severe CKD (67% versus 69%, *p* = 0.618 and 6% versus 9%, *p* = 0.229). Figure 1 depicts the process of patient selection in patients with severe CKD.

The final adjudicated diagnoses in the overall CKD population were NSTEMI type 1 in 156 (54%), NSTEMI type 2 in 55 (19%), unstable angina in 50 (17%), cardiac non coronary in 14 (5%), non-cardiac cause in 6 (2%), and unknown in 9 (3%) patients (Table 1). In the subgroup of patients undergoing revascularization, 156 (70%) patients had NSTEMI type 1, and 45 (20%) had NSTEMI type 2. In the conservatively treated group, the final adjudicated diagnosis was unstable angina in 50%, type 2 NSTEMI in 15%, and cardiac non-coronary in 13% of patients.

Baseline characteristics of CKD patients are summarized in Table S1 in the Supplementary Materials. The rates of males (74% versus 57%, *p* = 0.010), known CAD (75% versus 57%, *p* = 0.006), previous PCI (59% versus 44%, *p* = 0.036), and electrocardiogram abnormalities were significantly more frequent in the revascularization group (68% versus 52%, *p* = 0.018). The other demographic characteristics, cardiovascular risk factors, vital signs, left ventricular ejection fraction (LVEF), GRACE risk score (the Global Registry of Acute Coronary Events), and laboratory values at admission were equally distributed. Notably, 47% of patients had an eGFR between 30 and 15 mL/min/1.73 m^2^, and 46% were on dialysis for kidney failure, with similar rates among the revascularization groups.

### 3.2. Values of hs-cTnT during Serial Sampling

**Figure 2:**
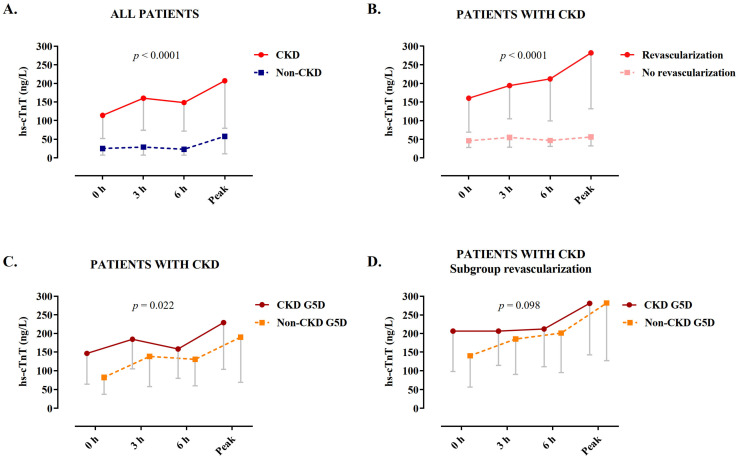
Levels of hs-cTnT at serial sampling. (**A**) Patients with normal renal function versus patients with severe CKD. (**B**–**D**) Among patients with severe CKD: (**B**) according to revascularization and conservative treatment, (**C**) in presence or absence of CKD G5D, and (**D**) in the subgroup of patients undergoing revascularization, in those with versus without CKD G5D. Data are depicted as medians with Q1. *p*-values are from a generalized linear model accounting for between and within group differences. CKD, chronic kidney disease; CKD G5D, chronic kidney disease G5 treated by dialysis; hs-cTnT, high-sensitivity cardiac troponin T.

Levels of hs-cTnT at presentation and serial sampling at 3 h and peak prior to angiography were significantly higher in patients with severe CKD as compared to the controls with normal renal function (median interquartile range (IQR) at presentation: 25 (7–102) versus 114 (52–314) ng/L, *p* < 0.001; at 3 h: 29 (7–116) versus 160 (74–369) ng/L, *p* < 0.001; at peak: 58 (11–210) versus 207 (79–537) ng/L, *p* < 0.001; Figure 2A and Supplementary Table S2). Within the cohort of patients with severe CKD, those undergoing revascularization had significantly higher levels of hs-cTnT compared to patients who were conservatively treated (at presentation: 46 (28–81) versus 160 (69–485) ng/L, *p* < 0.0001; at 3 h: 55 (29–88) versus 194 (105–409) ng/L, *p* < 0.001; at peak: 56 (32–93) versus 282 (132–746) ng/L, *p* < 0.001; Figure 2B and Supplementary Table S3). Within the cohort of patients with severe CKD, levels of hs-cTnT were significantly higher in those with CKD G5D (*p* = 0.022), but this difference was not significant in the subgroup of patients undergoing revascularization (*p* = 0.098, Figure 2C,D). Among patients treated conservatively, there was a significant inverse correlation between the levels of hs-cTnT at presentation and eGFR (correlation coefficient rS = 0.402, *p* = 0.001, Supplementary Figure S2).

### 3.3. Diagnostic Performance of Hs-cTnT in Patients with Severe CKD

**Figure 3:**
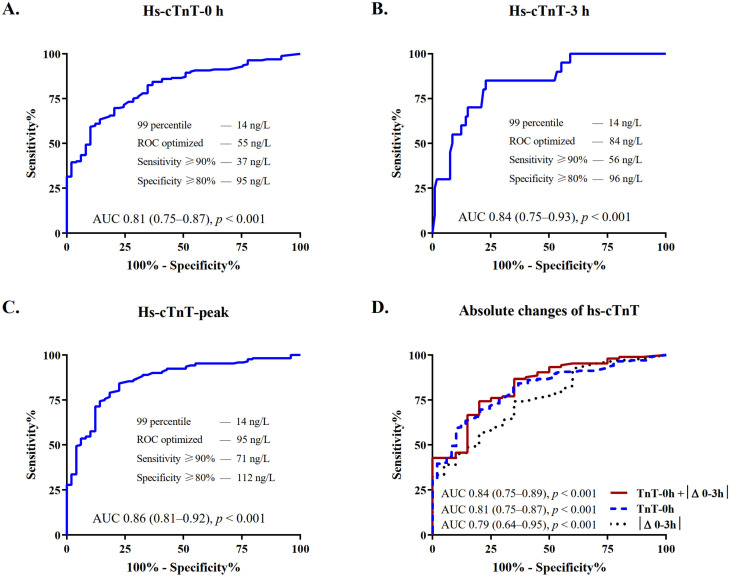
Diagnostic performance of hs-cTnT at serial sampling in patients with severe CKD. Depicted are ROCs and AUCs with 95% confidence intervals (CI) and predefined levels of sensitivity and specificity, describing the performance of the hs-cTnT assay for the diagnosis of NSTE-ACS requiring revascularization at (**A**) presentation, (**B**) 3 h, and (**C**) peak prior to angiography. (**D**) Depicts the improvement of the discrimination power of hs-cTnT at presentation when combined with absolute 3 h changes as compared to each of these variables alone. AUC, area under the receiver operating characteristic curve; CKD, chronic kidney disease; hs-cTnT, high-sensitivity cardiac troponin T; ROC, receiver-operating characteristic curve.

The diagnostic accuracy for the indication to revascularization was quantified by the AUC, with coronary angiography as reference exam. In patients with severe CKD, the AUC was high for measurements obtained at presentation (AUC, 95% confidence intervals (CI): 0.81 (0.75–0.87)) and increased further at 3 h and peak prior to angiography (0.84 (0.75–0.93) and 0.86 (0.81–0.92), respectively, Figure 3A–C and Supplementary Table S4).

**Figure 4:**
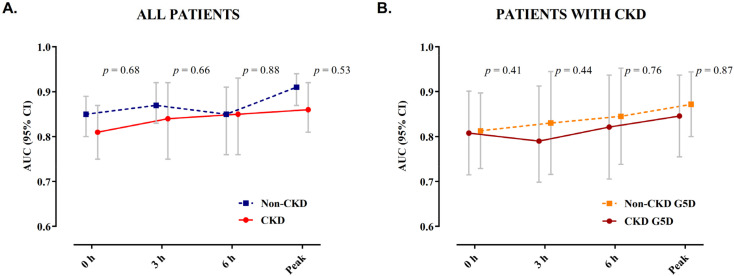
AUC of hs-cTnT during serial sampling. Depicted are the AUCs with 95% confidence intervals for hs-cTnT during serial sampling at presentation, 3 h, 6 h, and peak prior to coronary angiography in (**A**) patients with normal renal function and those with severe CKD and (**B**) among patients with severe CKD, comparing patients with CKD G5D to those with severe CKD but preserved diuresis. AUC, area under the receiver operating characteristic curve; CKD, chronic kidney disease; CKD G5D, chronic kidney disease G5 treated by dialysis.

Overall, the AUCs for patients with severe CKD were only slightly but not significantly lower when compared to patients with normal renal function. No significant difference in the diagnostic accuracy was observed in patients with CKD G5D compared to those with severe CKD but preserved diuresis (Figure 4).

Details on median absolute changes of hs-cTnT during serial sampling are shown in Table S5 in the Supplementary Materials. Patients undergoing revascularization had significantly higher early absolute changes as compared to those who did not (median |Δ0–3|: 34 versus 3 ng/L, *p* < 0.001). The diagnostic accuracy was good and increased from 0.79 (0.64–0.95) to 0.84 (0.75–0.89) when considering both hs-cTnT levels at presentation and absolute changes at 3 h (Figure 3D and Supplementary Table S5).

### 3.4. Optimal Cutoff Levels of Hs-cTnT for the Early Diagnosis of NSTE-ACS Requiring Revascularization

The optimal cutoff levels in patients with severe CKD were identified by ROC analysis (Figure 3). Table 2 depicts the diagnostic accuracy of hs-TnT in patients with severe CKD as compared to the conventional 99th percentile of the URL and to the controls with normal renal function. The assay sensitivity of the 99th percentile of 14 ng/L was high (98%), but this was associated with a marked decrease in specificity (10%). The optimal ROC-derived cutoffs of 55 ng/L at presentation and 95 ng/L at peak were, respectively, 3.9 and 6.8 times the level of hs-cTnT at the 99th percentile and 4.0 and 3.8 times the levels present in controls with normal renal function. The positive and negative predictive values (PPV and NPV) of the ROC optimized cutoff at presentation were higher compared to the 99th percentile (PPV, 95% CI: 89% (85–94) versus 79% (74–85); NPV, 95% CI: 52% (39–64) versus 50% (19–81)). Combining the ROC optimized cutoff levels for hs-cTnT at presentation and absolute 3 h changes, sensitivity increased to 98%; PPV and NPV further improved up to 93% and 86%, respectively (Table 2).

### 3.5. Outcomes at 30 Days and One Year of Follow-Up

At 30 days of follow-up, rates of MACE were significantly higher in patients requiring revascularization compared to patients receiving medical treatment only (11% versus 0%, *p* = 0.015). At a median follow-up of 365 days, 51 (29%) patients died (Supplementary Table S6). The rate of MACE was 42%, cardiovascular death 19%, MI 33%, and unplanned revascularization for ischemia 24%. All these events except cardiovascular death occurred significantly more frequently in the revascularization group. At one year, patients with hs-cTnT levels above these ROC-derived cutoffs were at higher risk of all-cause mortality (crude hazard ratio (HR), 95% CI at presentation: 2.73 (1.22–6.10), *p* = 0.012; at peak: 4.29 (1.70–10.9), *p* = 0.002) and MACE (at presentation: 2.73 (1.43–5.19), *p* = 0.001; at peak: 2.89 (1.52–5.51), *p* = 0.001). At multivariate analysis, the ROC-optimized cutoffs both at presentation and peak were independent predictors of all-cause mortality (HR, 95% CI at presentation: 2.36 (1.05–5.32), *p* = 0.03; at peak: 3.62 (1.42–9.22), *p* < 0.01), MI (at presentation: 4.03 (1.54–10.5), *p* < 0.01; at peak: 4.00 (1.56–10.3), *p* < 0.01), and MACE (at presentation: 3.19 (1.48–6.89), *p* < 0.01; at peak: 3.06 (1.43–6.54), *p* < 0.01), whereas CKD G5D was a predictor of all-cause mortality (HR, 95% CI: 2.09 (1.11–3.94), *p* = 0.02) and cardiovascular mortality (1.02 (1.31–7.55), *p* = 0.01, Supplementary Figure S4).

## 4. Discussion

To the best of our knowledge, this is the first study to investigate the diagnostic accuracy of hs-cTnT for the diagnosis of NSTE-ACS requiring revascularization in a considerable cohort of patients with severe CKD (eGFR < 30 mL/min/1.73 m^2^), including patients with CKD G5D. The following important findings emerged from our study: first, in patients with severe CKD, the hs-cTnT assay maintained a high diagnostic accuracy, which increased from presentation to later sampling points and when combined with early absolute changes. Second, the high diagnostic accuracy of the hs-cTnT assay was not reduced in patients undergoing dialysis for kidney failure. Third, hs-cTnT concentrations at presentation strongly and inversely correlated with the value of eGFR in patients not requiring revascularization. Fourth, in patients with severe CKD, the optimal cutoff for the diagnosis of NSTE-ACS requiring revascularization calculated at presentation was four times higher compared to patients with normal renal function. Fifth, the new identified ROC-optimized cutoffs were independent predictors of all-cause mortality, MACE, and MI at one-year follow-up.

Patients with varying degrees of CKD represent a common scenario seen in clinical practice; nonetheless, they have been systematically underrepresented or excluded from clinical trials and have been unthoroughly studied in the context of peri-MI. The cardiovascular risk increases with the degree of renal impairment and is ten to thirty times higher in patients with kidney failure treated by dialysis [29]. It is therefore of utmost importance to properly identify those who need an emergent revascularization and avoid the additional risks of an invasive procedure for those who do not. Particularly in the context of NSTE-ACS, large-scale registries have demonstrated better short- and long-term survival with early revascularization compared with drug therapy alone across all CKD stages [3,8,11,30,31], whereas in patients with stable coronary artery disease, advanced CKD, and moderate to severe ischemia, there was no survival benefit and no risk reduction in myocardial infarction for an initial invasive strategy compared with primary conservative therapy [32]. However, assessing the need for early revascularization in patients with severe CKD remains a major challenge, as in these patients, the most sensitive biomarker to date for the diagnosis of AMI, hs-cTnT, is chronically and often nonspecifically elevated [13,14]. The nature of this chronic elevation is incompletely understood and under investigation. Underlying mechanisms are probably multifactorial, including ongoing myocyte damage as a result of uremic toxicity, macrovasular or microvascular ischemia, anemia, and subordinately decreased renal clearance [33,34,35].

As expected, patients with severe CKD in our study population had significantly higher hs-cTnT values at all measuring time points compared to patients with normal renal function irrespective of the presence of NSTE-ACS. In fact, 96% of patients with severe CKD not requiring revascularization had elevated hs-cTnT above the 99th percentile of 14 ng/L, with an extremely low specificity of 10%. Among patients with severe CKD requiring revascularization, the median hs-cTnT values were significantly higher compared to patients who were not revascularized, which likely indicates that in presence of NSTE-ACS, a detectable increase of hs-cTnT levels is observed irrespective of the baseline hs-cTnT levels. In the present population with severe CKD, the AUC increased from 0.81 at presentation to 0.84 at 3 h and 0.86 at peak hs-cTnT prior to angiography, demonstrating a high diagnostic accuracy at serial sampling with no significant difference compared the control cohort with normal renal function. Furthermore, when combining the ROC-optimized cutoff levels for hs-cTnT at presentation and absolute 3 h changes, the PPV and NPV increased from 79 to 93% and from 50 to 86%, respectively, compared to the 99th percentile. Our results are in line with the published data from Twerenbold and colleagues [17]. Although the populations investigated differed among our studies with regards to the median eGFR (49 versus 23 mL/min/1.73 m^2^) and the prevalent stages of CKD (circa 90% CKD G3 versus 100% CKD G4 and G5), they could both demonstrate higher cutoff values of hs-cTnT and a high diagnostic accuracy. In a very recent study, the same authors investigated the performance of the 0/1 h algorithm in a similar population and concluded that although safety is high, the overall efficacy and specificity remain low despite modifications of the thresholds of the rule-in and rule-out [22]. Notably striking was the finding that of the included patients with CKD G4, only 1% ended up in the rule-out group, and 49% required further observation. A similar recent study from Kraus et al. [21] investigated the diagnostic accuracy of two hs-cTnT assays, demonstrating a better performance when using 2.5-fold changes in hs-cTnT levels at 3 h with a PPV of 0.8. Additionally, in this study, patients with severe CKD were underrepresented (3.8% with CKD G4 and 3.6% with CKD G5), and those undergoing dialysis were excluded. Despite excluding patients undergoing dialysis for kidney failure, these results underline the difficulties of balancing sensitivity and specificity in patients with CKD as compared to patients with normal renal function. Our study demonstrates that in patients with severe CKD, the threshold of the conventional 99th percentile derived from patients with normal renal function performs worse, as indicated by lower PPV and NPV compared to those identified in our study (79% versus 89% and 50% versus 52%, respectively).

The high diagnostic accuracy of hs-cTnT was maintained irrespective of the presence of CKD G5D. In line with the survey by Yang et al., we obtained higher ROC-derived cutoff levels for patients undergoing dialysis for kidney failure. In their study investigating optimal hs-cTnT cutoff levels in patients with renal dysfunction, the authors indicated that at various stages of CKD, including CKD G5D, different cutoff levels are needed despite the lack of a linear increase of the cutoff values with decreasing eGFR. The cutoff value was highest in patients undergoing dialysis and lowest in those with CKD G3. Unfortunately, no control cohort of patients with normal renal function was available, making it difficult to assess the overall assay performance in that community-based cohort.

In our study, coronary angiography was used as the most accurate exam to objectify outcomes in this diagnostically very challenging population. So far, there are only very few studies, all with major methodological issues, considering the presence of significant coronary stenosis at coronary angiography as a reference in comparison with different cTnT assays [36,37,38]. Of these, only the one by Ballocca et al. [36] applied an hs-cTnT assay, obtaining a poor AUC for the prediction of AMI in patients with chest pain and CKD. Most of the recent studies used clinical diagnosis of AMI without carrying out coronary angiography for all their patients [17,18,19,20,21,22]. The adjudication of AMI was based on hs-cTnT and its 99th percentile of healthy individuals together with a significant rise or fall in hs-cTnT. The definition of a significant rise or fall in hs-cTnT widely differed between the different investigations. While, for example, Twerenbold et al. applied an absolute change of at least 6 ng/L within three hours or 10 ng/L within six hours [22], Kraus et al. used a 20% rise or fall in the first six hours [21]. The latter survey even suggests a 250% change for their diagnostic algorithm. Furthermore, a strong inverse correlation between eGFR and absolute hs-cTnT changes could be demonstrated in patients with diagnoses other than NSTEMI, and patients with CKD were more than twice as likely to be diagnosed with NSTEMI and more than five times as likely to be diagnosed with NSTEMI type 2 [22]. In particular, the clinical distinction between NSTEMI type 1 and type 2 without having performed coronary angiography seems questionable in this collective. Thus, in these study cohorts with suspected ACS, all patients with an elevated baseline troponin and troponin dynamics related to predefined cutoffs on the kidney-healthy population would all have to be classified as true-positive cases (NSTEMI type 1 or 2) by definition and thus confirm the existing cutoffs. These studies investigating the diagnostic performance and optimal cutoffs of hs-cTnT for the diagnosis of AMI use hs-cTnT at the same time for the definition and adjudication of AMI. Hs-cTnT not only represents the object of investigation but also serves as a key part of the adjudication of the primary endpoint. Bearing in mind that patients with renal dysfunction often have chronic hs-cTnT elevations above the 99th percentile, atypical clinical presentation, and electrocardiogram abnormalities in the absence of AMI, this constitutes a methodological limitation. Finding an appropriate hs-cTnT cutoff in patients with severe CKD and suspected ACS who have undergone coronary angiography to objectify outcomes could help clarify the appropriate indication for cardiac catheterization and avoid further risks to these vulnerable patients.

Recent studies investigating the prognostic value of hs-cTnT more consistently observed a significant association of hs-cTnT with different outcome parameters, such as all-cause mortality and major adverse cardiovascular events [15,17,39,40]. In line with these findings, the cutoffs identified in our study were independent predictors of all-cause mortality, MI, and MACE. As previously reported, dialysis was an independent predictor of all-cause and cardiovascular mortality [4].

The strength of the present study is the use of catheterization as reference exam to independently adjudicate the need for revascularization and the inclusion of a considerable number of patients with severe CKD. Further, the inclusion of patients with CKD G5D increases the validity of the study by providing real-world data that comprise the whole spectrum of patients with severe CKD.

However, this study has limitations that merit consideration. Firstly, it was conducted in three national centers with a relatively small number of patients, although, to our knowledge, this is the largest cohort of patients with severe CKD being investigated for the optimal hs-cTnT cutoff. Therefore, the diagnostic accuracy of the cutoff value needs to be validated in larger cohorts from different geographical regions. Second, we only evaluated hs-cTnT values from a single, although widely used, assay; therefore, the present results are assay specific and cannot be applied to other assays without additional studies. Third, due to the fact that only patients with suspected NSTE-ACS undergoing coronary angiography have been included, our cohort has a higher pretest probability compared with the average clientele in the emergency department. Fourth, the indication for revascularization was based on coronary angiography only, and invasive tests for ischemia (i.e., by measurement of fractional flow reserve) were performed only in a fraction of patients. Lastly, serial hs-cTnT measurements were available for 84% of patients with severe CKD.

## 5. Conclusions

In patients with severe CKD and suspected ACS, the diagnostic performance of hs-cTnT to differentiate an acute myocardial injury due to presumed NSTE-ACS from a preexisting chronic one is improved by using higher assay-specific cutoff levels combined with early absolute changes.

## Figures and Tables

**Table 1:** Distribution of the adjudicated final diagnosis in patients with severe CKD.

	Revascularization			
	Total *n* = 290	No *n* = 68	Yes *n* = 222	*p*-Value
NSTEMI type 1	156 (53.8)	0 (0)	156 (70.3)	<0.01
NSTEMI type 2	55 (19.0)	10 (14.7)	45 (20.3)	0.38
Hypertensive crisis	32 (11.0) ^a^	7 (10.3) ^b^	25 (11.3) ^c^	1.00
Tachy-/bradyarrhythmia	28 (9.7) ^a^	7 (10.3) ^b^	21 (9.5) ^c^	0.82
Unstable angina	50 (17.2)	34 (50)	16 (7.2)	<0.01
Cardiac, non-coronary	14 (4.8)	9 (13.2)	5 (2.2)	0.001
Takotsubo syndrome	1 (0.3)	1 (1.5)	0 (0)	0.23
Pulmonary embolism	1 (0.3)	0 (0)	1 (0.5)	1.00
Endo-/myocarditis	4 (1.8)	0 (0)	4 (1.8)	0.58
Heart failure non-ischemic	8 (2.8)	8 (11.7)	0 (0)	<0.01
Non-cardiac cause	6 (2.1)	6 (8.8)	0 (0)	<0.01
Unknown	9 (3.1)	9 (13.2)	0 (0)	<0.01

Depicted are counts with frequencies (%). *p*-values are from Fisher’s exact test. CKD, chronic kidney disease; NSTEMI, non-ST-elevation acute myocardial infarction. ^a^ 5 patients, ^b^ 4 patients, and ^c^ 1 patient had both a hypertensive crisis and significant tachy-/bradyarrhythmia.

**Table 2:** Diagnostic performance of hs-cTnT in patients with severe CKD.

	Hs-cTnT Cutoff (ng/L)	Sensitivity (95% CI)	Specificity (95% CI)	PPV (95% CI)	NPV (95% CI)	Multiples of the 99 Percentile	Multiples of CKD vs. Control
0 h							
99th percentile ^a^	14	98 (95–99)	10 (2–19)	79 (74–85)	50 (19–81)	-	-
ROC optimized	55	83 (77–88)	65 (52–77)	89 (85–94)	52 (39–64)	3.9	4.0
Sensitivity ≥ 90%	37	90 (85–94)	48 (35–63)	86 (81–91)	57 (30–64)	2.6	2.7
Specificity ≥ 80%	95	70 (63–77)	80 (68–91)	92 (88–97)	43 (33–53)	6.8	7.0
Peak prior to angiography							
ROC optimized	95	84 (79–90)	78 (66–89)	93 (89–97)	59 (46–70)	6.8	3.8
Sensitivity ≥ 90%	71	90 (86–95)	60 (45–73)	89 (84–93)	63 (49–77)	5.0	2.8
Specificity ≥ 80%	112	79 (73–85)	82 (71–92)	94 (90–98)	53 (41–64)	8.0	4.5
0 h + |∆0 h–3 h|							
0 h ≥ 55 ng/L or		98 (91–100)	55 (25–82)	93 (83–97)	86 (42–99)		
3 h-change ≥ 4 ng/L							

Depicted are the results of the ROC analysis of hs-cTnT at presentation and peak prior to angiography in patients with severe CKD (eGFR < 30 mL/min/1.73 m^2^) compared to the controls with normal renal function and the 99th percentile. The discrimination increases when combining the ROC optimized cutoff values for hs-cTnT at presentation and absolute 3 h changes. eGFR, estimated glomerular filtration rate according to the CKD-EPI creatinine equation; hs-cTnT, high-sensitivity cardiac troponin T; NPV, negative predictive value; PPV, positive predictive value; ROC, receiver operating characteristic curve. ^a^ 99th percentile of the upper reference limit refers to the conventional assay-specific cutoff for the diagnosis of AMI in healthy individuals, as recommended in clinical practice guidelines.

## Data Availability

The data presented in this study are available on request from the corresponding author.

## Supplementary Materials

The following are available online at https://www.mdpi.com/article/10.3390/jcm10184216/s1, Figure S1: Flow chart of the selection process of patients with severe CKD included in the ROC analysis; Figure S2: Correlation between hs-cTnT and creatinine levels; Figure S3: Kaplan–Meier estimates of adverse events at one year; Figure S4. Independent predictors of adverse events at one year; Table S1: Baseline characteristics of patients with severe CKD; Table S2: Values of hs-cTnT during serial sampling in patients with normal renal function versus those with severe CKD; Table S3: Values of hs-cTnT during serial sampling in patients with severe CKD according to the indication for revascularization; Table S4: Diagnostic performance of hs-cTnT at serial sampling in patients with severe CKD; Table S5: Diagnostic performance at presentation and absolute changes of hs-cTnT in patients with severe CKD; Table S6: Outcomes at 30 days and one year of follow-up in patients with severe CKD.
