# Supplementary material for: High-Sensitivity Cardiac Troponin T in Patients with Severe Chronic Kidney Disease and Suspected Acute Coronary Syndrome
Source: J Clin Med. 2021 Sep 17;10(18):4216. doi: 10.3390/jcm10184216 (PMC8471888; doi:10.3390/jcm10184216)
Supplement: Supplementary file 1 [file jcm-10-04216-s001.zip › jcm-1353999-supplementary.pdf]

High-Sensitivity Cardiac Troponin T in Patients with Severe Chronic Kidney Disease and Suspected Acute Coronary Syndrome

SUPPLEMENTARY CONTENT

Table S1. Baseline characteristics of patients with severe CKD. 2

Table S2. Values of hs-cTnT during serial sampling in patients with normal renal function versus those with severe CKD. 4

Table S3. Values of hs-cTnT during serial sampling in patients with severe CKD according to the indication for revascularization. 5

Table S4. Diagnostic performance of hs-cTnT at serial sampling in patients with severe CKD. 6

Table S5. Diagnostic performance at presentation and absolute changes of hs-cTnT in patients with severe CKD. 7

Table S6. Outcomes at 30 days and one year of follow-up in patients with severe CKD. 8

Figure S1. Flow chart of the selection process of patients with severe CKD included in the ROC-analysis. 9

Figure S2. Correlation between hs-cTnT and creatinine levels. 10

Figure S3. Kaplan Meier estimates of adverse events at one year. 11

Figure S4. Independent predictors of adverse events at one year. 12

Table S1. Baseline characteristics of patients with severe CKD.

		Revascularization		
	**Total** **290**	**No** **68 (24)**	**Yes** **222 (76)**	**P-value**
Age	73 (64, 79)	74 (63, 80)	73 (64, 78)	0.780
Male	204 (70)	39 (57)	165 (74)	0.010
**Risk factors**				
BMI	26 (23, 31)	26 (23, 30)	26 (23, 31)	0.607
Hypertension	268 (92)	63 (93)	205 (92)	1.000
Hypercholesterolemia	213 (73)	49 (72)	164 (74)	0.756
Diabetes mellitus	162 (56)	31 (46)	131 (59)	0.069
Current smoking	69 (32)	13 (28)	56 (33)	0.597
COPD	55 (19)	14 (21)	41 (18)	0.725
Family history	33 (39)	4 (25)	29 (42)	0.263
**History**				
CAD	205 (71)	39 (57)	166 (75)	0.006
1-vessel CAD	22 (8)	6 (9)	16 (7)	0.795
2-vessel CAD	32 (11)	6 (9)	26 (12)	0.517
3-vessel CAD	142 (51)	27 (40)	115 (54)	0.051
Previous MI	157 (54)	30 (44)	127 (57)	0.071
Previous PCI	162 (56)	30 (44)	132 (59)	0.036
Previous CABG	95 (33)	17 (25)	78 (35)	0.140
Previous stroke	43 (15)	8 (12)	35 (16)	0.559
PAD	110 (38)	20 (29)	90 (41)	0.116
**Vital signs**				
Systolic BP	139 (120, 156)	138 (117, 156)	140 (120, 157)	0.614
Diastolic BP	71 (62, 83)	76 (66, 85)	70 (61, 82)	0.128
Heart rate	80 (71, 94)	80 (69, 90)	80 (72, 95)	0.416
SaO_2	97 (95, 99)	97 (95, 100)	97 (95, 99)	0.430
Angina CCS Class	4 (3, 4)	3 (3, 4)	4 (3, 4)	0.06
EGC changes	179 (64)	34 (52)	145 (68)	0.018

ST-segment depression	82 (29)	10 (15)	72 (34)	0.003
T-wave inversion	84 (30)	17 (26)	67 (32)	0.443
LBBB	34 (12)	8 (12)	26 (12)	1.000
Ns ST-segment elevations	18 (6)	3 (5)	15 (7)	0.578
LVEF	47 (35, 55)	54 (34, 60)	45 (35, 55)	0.076
GRACE risk score	135 (118, 154)	131 (111, 150)	136 (119, 155)	0.167
**Renal function**				
Creatinine, mg/dl	3.2 (2.4, 5.7)	3.0 (2.2, 5.0)	3.3 (2.4, 5.7)	0.176
eGFR, mL/min/1.73m^2	23 (18, 27)	24 (18, 28)	23 (18, 27)	0.499
**Stages of CKD**				
eGFR 15-29	137 (47)	36 (53)	101 (45)	0.331
eGFR <15	19 (7)	5 (7)	14 (6)	0.781
CKD G5D	134 (46)	27 (40)	107 (48)	0.266
**Intervention**				
PCI	197 (68)	0 (0)	197 (89)	<0.001
CABG	25 (9)	0 (0)	25 (11)	<0.001

Depicted are medians with interquartile ranges (IQR) or counts with frequencies (%). P-values are from Wilcoxon rank sum or Fisher´s Chi2 tests. Severe CKD defined as eGFR <30 mL/min/1.73m^2. BMI: body mass index in kg/m2; BP: blood pressure; CABG: coronary artery bypass grafting; CAD: coronary artery disease; CCS: Canadian cardiovascular society; CKD: chronic kidney disease; COPD: chronic obstructive pulmonary disease; CKD G5D: chronic kidney disease G5 treated by dialysis; eGFR: estimated glomerular filtration rate according to the CKD-EPI creatinine equation; GRACE: the Global Registry of Acute Coronary Events; LBBB: left bundle-branch block, LVEF: left ventricular ejection fraction; MI: myocardial infarction; ns: non significant; PAD: peripheral artery disease; PCI: percutaneous coronary intervention; SaO2: arterial oxygen saturation.

Table S2. Values of hs-cTnT during serial sampling in patients with normal renal function versus those with severe CKD.

**Hs-cTnT (ng/L)**	**Normal renal function** n=300	**Severe CKD** n=290	**P-value**
**Presentation**	25 (7-102)	114 (52-314)	<0.01
**3 hours**	29 (7-116)	160 (74-369)	<0.01
**Peak**	58 (11-210)	207 (79-537)	<0.01

Depicted are medians with interquartile ranges. P-values are from Wilcoxon rank sum test. Severe CKD defined as eGFR <30 mL/min/1.73m^2. CKD: chronic kidney disease; eGFR: estimated glomerular filtration rate according to the CKD-EPI creatinine equation; hs-cTnT: high sensitivity cardiac troponin T.

Table S3. Values of hs-cTnT during serial sampling in patients with severe CKD according to the indication for revascularization.

	Revascularization		
**Hs-cTnT (ng/L)**	**No**<br>n=68	**Yes**<br>n=222	**P-value**
**Presentation**	46 (28-81)	160 (69-485)	<0.001
**3 hours**	55 (29-88)	194 (105-409)	<0.001
**6 hours**	47 (31-96)	212 (99-572)	<0.001
**Peak**	56 (32-93)	282 (132-746)	<0.001

Depicted are medians with interquartile ranges. P-values are from Wilcoxon rank sum test. Severe CKD defined as eGFR <30 mL/min/1.73m^2. CKD: chronic kidney disease; eGFR: estimated glomerular filtration rate according to the CKD-EPI creatinine equation; hs-cTnT: high sensitivity cardiac troponin T.

Table S4. Diagnostic performance of hs-cTnT at serial sampling in patients with severe CKD.

	Hs-cTnT-0h	Hs-cTnT-3h	Hs-cTnT-peak
**AUC (95% CI)**	0.81 (0.75-0.87)	0.84 (0.75-0.93)	0.86 (0.81-0.92)
**ROC optimized cutoff**	≥ 55 ng/L	≥ 84 ng/L	≥ 95ng/L
Sensitivity (95% CI)	83 (77-88)	85 (76-91)	84 (79-90)
Specificity (95% CI)	65 (52-77)	70 (46-87)	78 (66-89)
**99th percentile**	≥ 14 ng/L	≥ 14 ng/L	≥ 14 ng/L
Sensitivity (95% CI)	98 (95-99)	99 (94-100)	98 (95-100)
Specificity (95% CI)	10 (2-19)	15 (4-39)	4 (1-15)
**Specificity optimized cutoff (≥ 80%)**	≥ 95 ng/L	≥ 96 ng/L	≥ 112 ng/L
Sensitivity (95% CI)	70 (63-77)	77 (68-85)	79 (73-85)
Specificity (95% CI)	80 (68-91)	85 (61-96)	82 (71-92)
**Sensitivity optimized cutoff (≥ 90%)**	≥ 37 ng/L	≥ 56 ng/L	≥ 71 ng/L
Sensitivity (95% CI)	90 (85-94)	91 (84-96)	90 (86-95)
Specificity (95% CI)	49 (35-63)	55 (32-76)	60 (45-73)

Depicted are AUCs and different cutoff levels with sensitivities and specificities in percent and 95% confidence intervals (CI) of hs-cTnT at presentation, 3 hours and peak prior to angiography. Severe CKD defined as eGFR <30 mL/min/1.73m^2. AUC: area under the receiver operating characteristic curve; CKD: chronic kidney disease; eGFR: estimated glomerular filtration rate according to the CKD-EPI creatinine equation; ROC: receiver operating characteristic curve.

Table S5. Diagnostic performance at presentation and absolute changes of hs-cTnT in patients with severe CKD.

	Hs-cTnT-0h	│Δ0h-3h│	│Δ0h-6h│	0h + │Δ3-0h│
**Median (IQR)**	130 (48, 411)	27 (4, 108)	65 (7, 301)	-
**AUC (95% CI)**	0.81 (0.75-0.87)	0.79 (0.64-0.95)	0.84 (0.72-0.96)	0.84 (0.75-0.89)
**ROC optimized cutoff**	≥ 55 ng/L	≥ 4 ng/L	≥ 8 ng/L	0h ≥ 55 ng/L or │Δ0h-3h│≥ 4 ng/L
Sensitivity (95% CI)	83 (77-88)	91 (81-96)	84 (70-92)	98 (91-100)
Specificity (95% CI)	65 (52-77)	64 (32-88)	70 (35-92)	55 (25-82)
**PPV (95% CI)**	89 (85-94)	94 (84-98)	93 (80-98)	93 (83-97)
**NPV (95% CI)**	52 (39-64)	54 (26-80)	47 (22-73)	86 (42-99)

Depicted are medians in ng/L with interquartile ranges (IQR) and AUCs with 95% confidence intervals (95% CI) of hs-cTnT at presentation, absolute changes at 3 and 6 hours and the combination of hs-cTnT at presentation with absolute 3-hour changes. Severe CKD defined as eGFR <30 mL/min/1.73m^2. AUC: area under the curve; CKD: chronic kidney disease; eGFR: estimated glomerular filtration rate according to the CKD-EPI creatinine equation; NPV: negative predictive value; PPV: positive predictive value.

Table S6. Outcomes at 30 days and one year of follow-up in patients with severe CKD.

		Revascularization		
	Total	No	Yes	P-value
**30 days**				
All cause death	14 (7)	1 (2)	13 (8)	0.199
MACE	17 (8)	0 (0)	17 (11)	0.015
Cardiovascular death	9 (4)	0 (0)	9 (6)	0.214
Myocardial infarction	9 (5)	0 (0)	9 (6)	0.120
Unplanned revascularization	6 (3)	0 (0)	6 (4)	0.339
**365 days**				
All cause death	51 (29)	5 (13)	46 (34)	0.010
MACE	71 (42)	4 (11)	67 (50)	<0.001
Cardiovascular death	29 (19)	3 (8)	26 (23)	0.056
Myocardial infarction	49 (33)	1 (3)	48 (41)	<0.001
Unplanned revascularization	33 (24)	2 (6)	31 (31)	0.002

Depicted are counts with frequencies (%). P-values are from log-rank tests. Severe CKD defined as eGFR <30 mL/min/1.73m². CKD: chronic kidney disease; eGFR: estimated glomerular filtration rate according to the CKD-EPI creatinine equation; MACE: major adverse cardiovascular events (cardiovascular death, myocardial infarction, unplanned revascularization).

Figure S1. Flow chart of the selection process of patients with severe CKD included in the ROC-analysis.

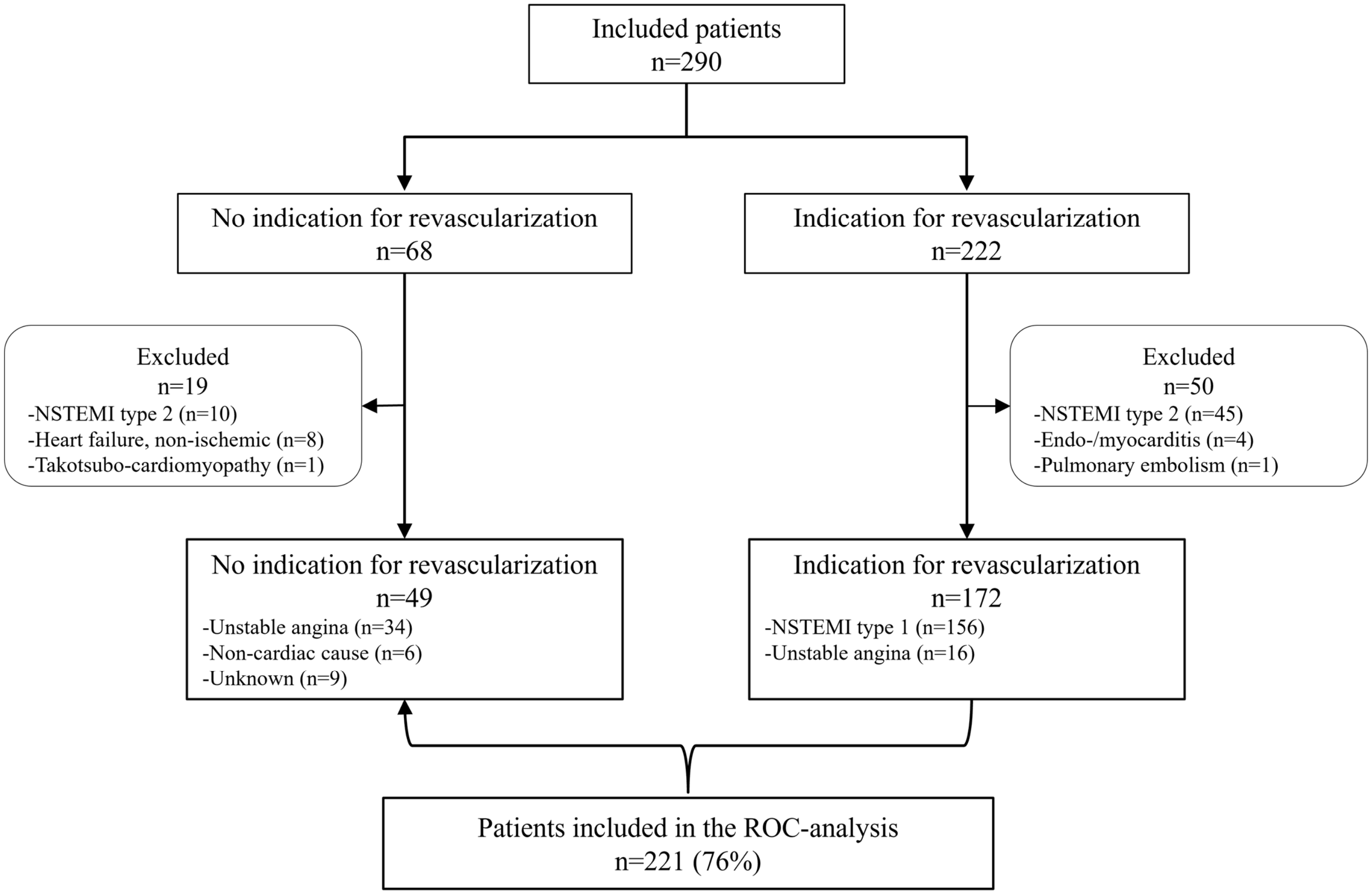

Severe CKD defined as eGFR <30 mL/min/1.73m². CKD: chronic kidney disease; eGFR: estimated glomerular filtration rate according to the CKD-EPI creatinine equation; NSTEMI: non-ST-elevation acute myocardial infarction; ROC: receiver operating characteristic curve.

Figure S2. Correlation between hs-cTnT and creatinine levels.

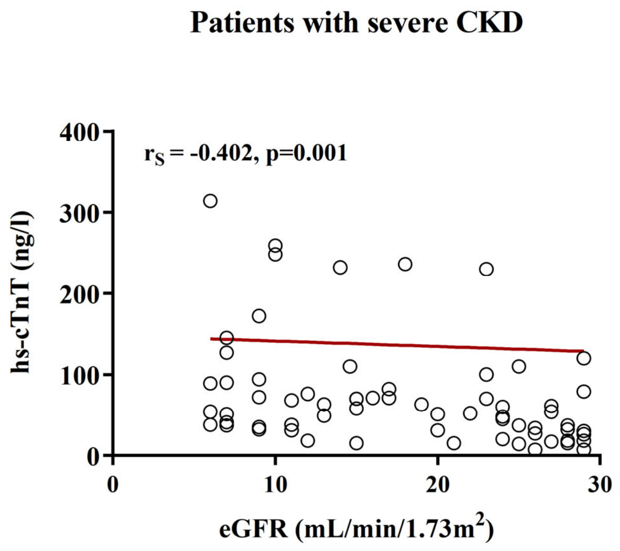

Depicted is the correlation between levels of hs-cTnT and creatinine in the non-revascularization subgroup of the cohort with severe CKD. Severe CKD defined as eGFR <30 mL/min/1.73m². RS is the Spearman correlation coefficient. CKD: chronic kidney disease; eGFR: estimated glomerular filtration rate according to the CKD-EPI creatinine equation.

Figure S3. Kaplan Meier estimates of adverse events at one year.

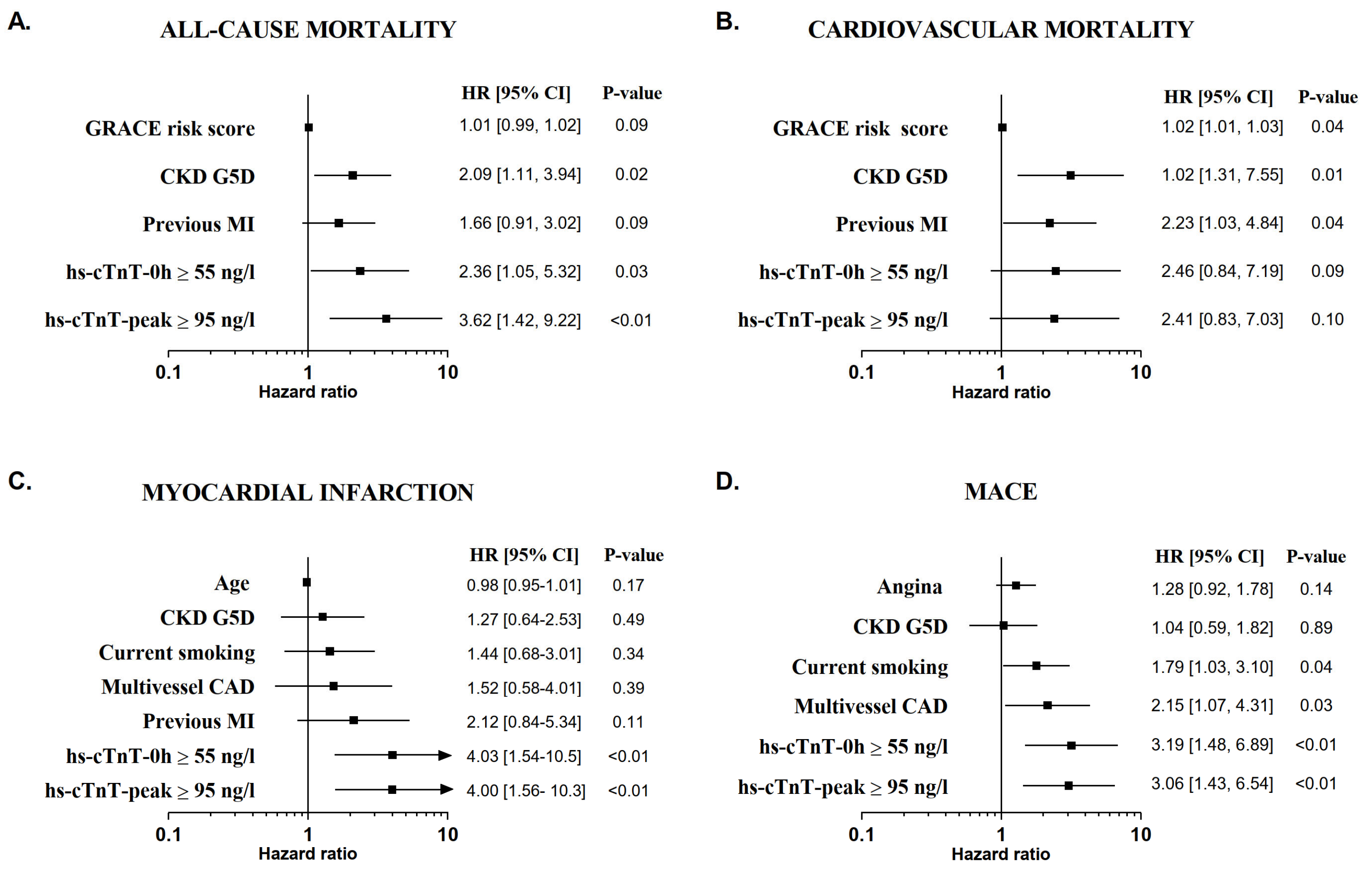

Depicted are the Kaplan Meier estimates of adverse events in patients with severe CKD (eGFR <30 mL/min/1.73m²) at one year, according to ROC optimized hs-cTnT cutoffs at presentation and peak of all-cause mortality (A and C) and MACE (B and D). Cumulative incidence (%) of events is from Kaplan Meier, crude hazard ratios (HR) and 95% CIs are from Cox regressions. CKD: chronic kidney disease; eGFR: estimated glomerular filtration rate according to the CKD-EPI creatinine equation; hs-cTnT: high sensitivity cardiac troponin T; MACE: composite of cardiovascular death, myocardial infarction or unplanned revascularization for ischemia; ROC: receiver-operating characteristic curve.

Figure S4. Independent predictors of adverse events at one year

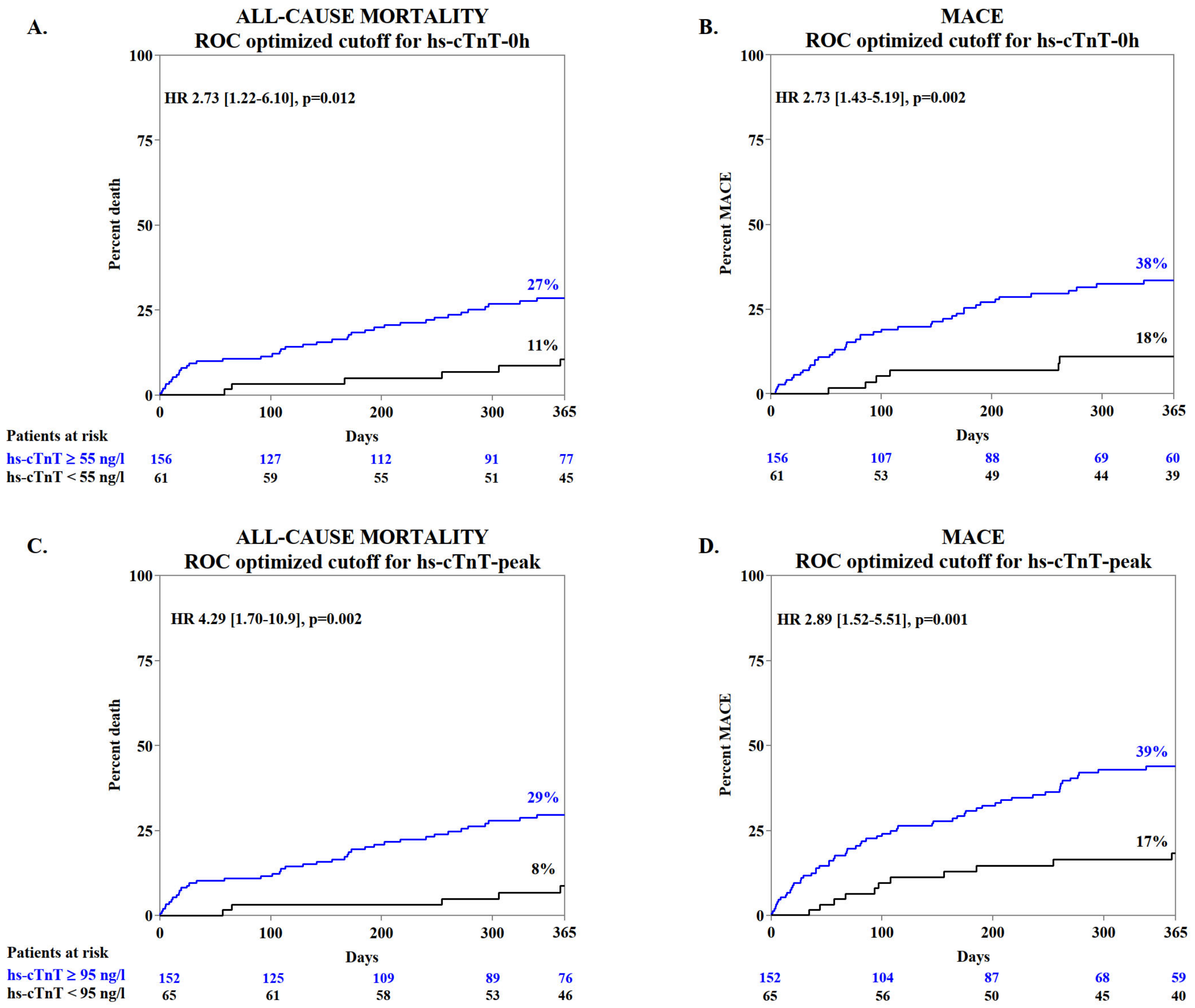

Depicted are the independent predictors of adverse events in patients with severe CKD (eGFR <30 mL/min/1.73m²) at one year: A) all-cause mortality, B) cardiovascular mortality, C) myocardial infarction and D) MACE. The symbols indicate the point estimate (hazard ratios [HR]) and the lines the 95% Confidence Intervals [95% CI] from multivariate logistic regression. The arrow indicates a CI-value beyond the shown axis limit. CAD: coronary artery disease; CKD: chronic kidney disease; eGFR: estimated glomerular filtration rate according to the CKD-EPI creatinine equation; CKD G5D: chronic kidney disease G5 treated by dialysis; GRACE: Global Registry of Acute Coronary Events; MI: myocardial infarction; MACE: composite of cardiovascular death, MI or unplanned revascularization for ischemia; ROC: receiver-operating characteristic curve.
